# Epilepsy Genetics and Precision Medicine in Adults: A New Landscape for Developmental and Epileptic Encephalopathies

**DOI:** 10.3389/fneur.2022.777115

**Published:** 2022-02-17

**Authors:** Álvaro Beltrán-Corbellini, Ángel Aledo-Serrano, Rikke S. Møller, Eduardo Pérez-Palma, Irene García-Morales, Rafael Toledano, Antonio Gil-Nagel

**Affiliations:** ^1^Epilepsy Program, Neurology Department, Hospital Ruber Internacional, Madrid, Spain; ^2^Department of Epilepsy Genetics and Personalized Treatment, The Danish Epilepsy Centre, Dianalund, Denmark; ^3^Universidad del Desarrollo, Centro de Genética y Genómica, Facultad de Medicina Clínica Alemana, Santiago, Chile; ^4^Epilepsy Unit, Neurology Department, Clínico San Carlos University Hospital, Madrid, Spain; ^5^Epilepsy Unit, Neurology Department, Ramón y Cajal University Hospital, Madrid, Spain

**Keywords:** neurogenetics, precision therapy, seizure, personalized medicine, rare diseases, genetic testing, intellectual disability, diagnostic yield

## Abstract

This review aims to provide an updated perspective of epilepsy genetics and precision medicine in adult patients, with special focus on developmental and epileptic encephalopathies (DEEs), covering relevant and controversial issues, such as defining candidates for genetic testing, which genetic tests to request and how to interpret them. A literature review was conducted, including findings in the discussion and recommendations. DEEs are wide and phenotypically heterogeneous electroclinical syndromes. They generally have a pediatric presentation, but patients frequently reach adulthood still undiagnosed. Identifying the etiology is essential, because there lies the key for precision medicine. Phenotypes modify according to age, and although deep phenotyping has allowed to outline certain entities, genotype-phenotype correlations are still poor, commonly leading to long-lasting diagnostic odysseys and ineffective therapies. Recent adult series show that the target patients to be identified for genetic testing are those with epilepsy and different risk factors. The clinician should take active part in the assessment of the pathogenicity of the variants detected, especially concerning variants of uncertain significance. An accurate diagnosis implies precision medicine, meaning genetic counseling, prognosis, possible future therapies, and a reduction of iatrogeny. Up to date, there are a few tens of gene mutations with additional concrete treatments, including those with restrictive/substitutive therapies, those with therapies modifying signaling pathways, and channelopathies, that are worth to be assessed in adults. Further research is needed regarding phenotyping of adult syndromes, early diagnosis, and the development of targeted therapies.

## Introduction

The field of epilepsy genetics has emerged in clinical practice and is rapidly evolving in the last years (1). Within the scope of genetic epilepsies, the main group of entities where a genetic etiology can be found are the developmental and epileptic encephalopathies (DEEs), defined as wide electroclinical syndromes characterized by epilepsy, developmental delay or regression, or intellectual disability, an abnormal EEG and other possible neurological or systemic manifestations (2). While DEEs have predominantly a pediatric onset, most patients will reach adulthood, frequently undiagnosed (3). DEEs are rare and clinically heterogeneous. Understanding the complete landscape of disease presentation and trajectories over time is needed to accurately manage expectations, model disease outcome, comorbidities, and prognosis. Most of our acquired knowledge comes from the study of pediatric DEE patients, but several cohorts of adults with DEEs of diverse genetic origin have also been reported, even some adult-onset DEE case reports (4). Natural history studies are a valuable source of information able to systematically assess the clinical evolution of DEE patients. However, they are expensive and slow (5). For this reason, neurologists assessing adult patients should include these conditions and genetic testing in their daily clinical practice.

Beyond the conventional symptomatic therapeutic approaches, the key for precision medicine lies in unveiling the specific etiopathology of the DEE in each patient (structural, infectious, immune-mediated, metabolic, genetic, or unknown) (2, 6). The present common denominator of all these etiologies is an immense phenotypical heterogeneity, with some exceptions (7). Nowadays, most challenging etiologies to identify after a basic diagnostic workup (including anamnesis, physical examination, neuroimaging, EEG and basic and metabolic blood, urine and cerebrospinal fluid -CSF- tests), are genetic and unknown, which is likely meant to be also genetic in its majority (8).

In these lines, traditional electroclinical entities such as Ohtahara, West or Lennox-Gastaut syndromes, among others, could be currently considered under the wide definition of DEEs, with further particular delimitating traits, but again displaying the same broad range of etiological possibilities (7).

This narrative review aims to provide an updated perspective of etiology, clinical presentation, diagnostic workup and management of adult patients with DEEs, with a special focus on those with genetic origin, covering controversial issues, such as who are the optimal candidates for genetic testing, which genetic tests to request and how to interpret them.

## Methods

A literature review was carried out using the following terms indexed in the thesaurus of Medline/Pubmed: “Epilepsy AND genetics AND adult” (last accessed in June, 2021). Results were filtered by relevance for the topic by a team of experienced epileptologists with expertise in the management of genetic epilepsies and DEEs. Additionally, the contents and recommendations presented in this review are also based on the authors' published and unpublished experience in the diagnosis and management of genetic epilepsies in adult patients.

## Basic Concepts About Types of Genetic Variants

Before addressing the etiology, clinical presentation, diagnostic workup and therapy of adults with DEEs of genetic origin, it is necessary to briefly remind the principal types of genetic variants (9) that take part in the etiopathology of these entities.

- Single Nucleotide Variants (SNVs): a single nucleotide in a DNA sequence is substituted with a different type of nucleotide. If the SNV is located within the coding region of a gene, the SNVs can be further classified into *synonymous* (no amino acid change and usually benign, unless they affect a splicing site); *missense* (change of one amino acid within the protein sequence), or *nonsense* (the nucleotide change results in a premature stop codon, and usually non-functional protein product). SNVs are the most frequent pathogenic variants in genetic epilepsies (10).
- Indels: small (between 1 and 49 base pairs –bp- in length) *insertions* or *deletions* in a DNA sequence. If located within the coding sequence of a gene, a number of amino acids will be added to or deleted from the original protein. If the length of the indel is not a multiple of 3, it will result in a frameshift variant, disrupting the reading frame of all the following bases. Changes in the reading frame usually lead to downstream premature stop codons.
- Structural variants: *insertion, deletion, duplication, translocation* or *inversion* of segments of DNA from 50 bp up to millions of bp in length, even reaching chromosomal scales. Specifically, increments or reductions of the numbers of copies of a particular gene or DNA sequence in these length terms are referred to as *copy number variants* (CNVs), being the most frequent pathogenic structural variant in epilepsies of genetic origin (up to 16% in some series) (11). There are even larger structural abnormalities. For example, the occurrence of one or more extra or missing chromosomes is known as aneuploidy.
- Nucleotide repeat expansions: increment of the number of adjacent repetitions of a determined nucleotide (e.g., triplets) in a given DNA region, leading to different functional results depending on the number of repetitions and other factors. These variants are related to particular syndromes, such as familial cortical myoclonus (12) or Fragile X syndromes (13) and cannot be detected with conventional methods such as gene-panel sequencing or microarrays (14).

Concerning the above-mentioned variants, the diverse structural changes in proteins may lead to different grades of functional disruption depending on additional factors (15). Particularly, regarding channelopathies, SNVs could result in both, a loss or a gain of function of the affected ion channel, entailing therapeutic considerations (even age-related, as in *SCN2A*-related encephalopathies) (16). Furthermore, various types of alterations in non-coding and intronic regions (including the intron-exon boundaries and their mutations, which could potentially result in splicing disorders including poison exons) (17, 18), as well as epigenetic variations (19) (e.g., in Angelman syndrome) (20), have emerged in the last decades and should not be dismissed as potentially disease-causing mechanisms. Along these lines, beyond monogenic epilepsies, oligo- and polygenic substrates are emerging as phenotype-modifying factors in genetic epilepsies, converging in the latest polygenic risk scores (21, 22).

Finally, certain concepts with respect to patterns of inheritance are also worthy of a quick reminder (9). Depending on the affected cell tissue, mutations can occur in the sperm or the eggs (germ-line mutations that might be inherited by the offspring) or can occur in the rest of the body cells (somatic mutations that usually are not heritable) (23, 24). Somatic mutations intersect with the concept of mosaicism, which refers to a condition in which just a determined percentage of particular somatic cell lines of an individual carries the target genetic variant.

Concerning segregation studies, a contrast between parental-inherited variants and *de novo* variants (alterations newly occurring in an individual, as a result of a change in a germ cell or fertilized egg) is usually established. Of note, the most frequent and clearly established mechanism in the origin of genetic DEEs is *de novo* pathogenic variants (10). However, a small but significant percentage of cases that come from transmissible parental germline mosaicisms could be mistaken as *de novo* variants depending on the employed technique, resulting in relevant implications for genetic counseling (25).

## Basic Concepts About Types of Genetic Testing Techniques

Major genetic testing techniques and their coverage regarding the mentioned types of variants (10, 26–29) is warranted (Table 1). Another technique for detection of indels and other small structural variants, as well as certain epigenetic variation is known as multiplex ligation-dependent probe amplification (MLPA) (34). As mentioned, the detection of these kind of variants is being progressively encompassed, with reasonable accuracy, within NGS techniques and CGH-arrays, sometimes using MLPA for validation of these variants.

**Table 1:** Different genetic testing techniques and their coverage.

	**Aneuploidies**	**Balanced translocations/inversions**	**Unbalanced translocations**	**Copy number variants (CNVs)**	**Rest of structural variants**	**Nucleotide repeat expansions**	**Indels**	**Single nucleotide variant (SNVs)**	**Variations in non-coding regions/intronic regions**	**Epigenetic variations**	**Mitochondrial Genome**
Karyotype	YES	YES	YES (if large enough)	YES (if large enough)	YES (if large enough)	NO	NO	NO	NO	NO	NO
Fluorescence *in situ* hybridization (FISH)	YES	YES	YES (if known)	YES	YES	NO	NO	NO	NO	NO	NO
Comparative genomic hybridization-array (CGH-array)	YES	NO	YES	YES	YES	NO	NO	NO	Depends^A^	NO	NO
Gene Panel	NO	NO	NO	Depends^B^	Depends^B^	NO	YES	YES	NO	NO	NO
Whole exome sequencing (WES)	YES	NO	YES	Depends^C^	Depends^C^	NO^D^	YES	YES	NO^E^	NO	Depends^F^
Whole genome sequencing (WGS)	YES	YES	YES	YES	YES	NO^D^	YES	YES	YES	NO	Depends^F^

*A: depending on the target and extension of the probes. B, C: to be specifically checked regarding each laboratory. In the last years, newer bioinformatic tools allowing structural variants analysis basing on next generation sequencing (NGS) techniques (gene panels, whole exome sequencing –WES- and whole genome sequencing -WGS) are being implemented. In the case of gene panels (B), analysis would be restricted to the exons of the predetermined genes (n~100–300), whereas concerning WES (C), analysis would include the whole exome (n > 18.000) (30, 31). D: additional bioinformatic algorithms are currently under development in order to detect this kind of variants basing on NGS techniques (32). E: except variants present in the intron-exon boundaries, which WES detects. F: to be specifically checked regarding each laboratory (newer bioinformatic tools allowing analysis of mitochondrial genome basing on WES and WGS are being implemented) (33)*.

Some considerations need to be addressed regarding these types of genetic testing techniques. Firstly, analysis of nucleotide repeat expansions would require a different specific study (triplet-primed polymerase chain reaction –PCR- with specific primers) (35), although further bioinformatic tools are under development to allow their analysis basing on next generation sequencing (NGS) techniques [gene panels, whole exome sequencing (WES) and whole genome sequencing -WGS] (32). Examination of non-coding regions or intronic regions would also require distinct procedures within gene panels or WES, or a WGS (17), except variants present in intron-exon boundaries, whose detection would not require additional processing Analysis of epigenetic variations would as well involve a separated technique (34, 36). Besides, detection of mosaicism when employing NGS techniques would necessitate a deep sequencing approach (coverage > 100x) when suspected (inferior coverages would probably lead to misdetection of the mosaic variant and, for instance, a mistaken label of *de novo* in segregation studies) (37, 38).

## Genotype-Phenotype Correlation

Phenotype of DEEs includes a variable combination of epilepsy types (diverse classical and non-classical syndromes), developmental delay or regression, or intellectual disability, an abnormal EEG, and other possible neurological or systemic manifestations (movement disorders, non-epileptic paroxysmal disorders, sleep disturbances, dysautonomia, behavior disorders, dysmorphias, MRI abnormalities, or other alterations) (2).

Regarding DEEs of genetic origin, these elements may be present in different combinations and grades in each patient, depending on still scarcely defined genetic and environmental factors. Further, the phenotype observed at clinical presentation in pediatric ages is not a static picture, but dynamic in time. The above-mentioned compounding domains may vary along late infancy, adolescence, and adulthood, fluctuating in grade and importance, and even disappearing or appearing for the first time in the natural history of the disease (3, 7).

This concept is best exemplified in Dravet syndrome. In this entity, the well-known epilepsy-predominant clinical picture presenting at 5–8 months of life and consisting mainly of febrile and afebrile generalized clonic or hemiclonic seizures, gives way to a different setting in adolescence and adulthood (39, 40). Older patients will manifest less epileptic burden, predominantly generalized tonic-clonic seizures during sleep (41), and display other prevailing features, such as cognitive, behavioral and complex motor deficits, or even Parkinsonian traits and dysautonomia (42). Although still barely described for most conditions within this group, this phenotypical evolution is emerging as a general attribute of genetic DEEs, and should be taken into account by adult-patient neurologists, since the patients they assess may not resemble the ones described in neuropediatric series.

On the other hand, genotype-phenotype correlations in this context are still poor. Circumscribed to the general DEE phenotype, different genes showing diverse genetic alterations giving rise to proteins with several types and grades of dysfunction, could converge in the same electroclinical syndrome (genotypical heterogeneity) (15). The opposite could also be applied, different electroclinical syndromes could be caused by the same genetic alteration in two different individuals (phenotypical heterogeneity) (43).

Moreover, within this framework of poor correlations, given a particular genetic variant, the range of severity of the subsequent syndrome could vary from mild epilepsy to devastating encephalopathies. Again, extensively studied *SCN1A* pathogenic variants provide a good model for this concept, since similar variants could lead both to a Dravet syndrome phenotype and to a Generalized Epilepsy with Febrile Seizure plus phenotype (GEFS+) in two different subjects (44). Although most literature is biased toward more severe cases because genetic tests are mostly requested in this subgroup, emerging milder phenotypes should be taken into consideration by clinicians since their diagnosis will have management implications.

Up to present, research efforts have tried to adapt clinical syndromes to genetic etiologies with modest results. Nevertheless, newer approaches such as deep and reverse phenotyping have arisen in the last years. In this vein, current investigations focus on profoundly describing the phenotype of a given genetic variant in larger samples of patients (45). This approach is giving rise to a change in nomenclature, and progressively allowing the depiction of increasingly more etiology-specific syndromes, for instance *CDKL5* (46), *KCNQ2* (47), and *STXBP1* (48)-related disorders, or even entities with better genotype-phenotype correlations, such as *PCDH19*-related DEE (49). Other worth-to-consider well-known exceptions to these poor genotype-phenotype correlations are the already-mentioned Dravet syndrome, tuberous sclerosis complex (50) or Rett syndrome (51), among others.

## Adult Patients with DEEs: Who to Test

With respect to the selection of candidates for genetic testing and the diagnostic yield of different techniques, four main series have been published in the last years, in addition to other previous reports (52, 53), differing in their inclusion/exclusion criteria and their initial diagnostic approaches. Further details regarding the main findings of these series are displayed in Table 2.

**Table 2:** Main series examining the diagnostic yield and results of diverse genetic testing techniques in adult patients with DEEs or epilepsy and intellectual disability.

**References**	***N***	**Target phenotype**	**Diagnostic yield**	**Type of variant**	**Inheritance**	**Factors increasing the probability of genetic diagnosis**	**Percentage of patients that benefited from diagnostic-related changes in management**
Minardi et al. (54)	71 adults	DEEs of unknown etiology	WES: 25.3%	SNVs in 83.3% of the diagnosed patients (66.7% of them missense)	70.8% were novel or *de-novo* variants (2 AD and 4 AR among the inherited)	Brain MRI malformations, early onset epilepsy or dysmorphisms.	50% of diagnosed patients
Benson et al. (37)	74 adults (and 27 children)	Epilepsy and intellectual disability of unknown etiology	Trio-WES: 30% of adults	SNVs in 85% of the diagnosed adults (63.6% of them non-synonymous)	70% of the diagnosed adults displayed *de-novo* variants (4 AD, 2 AR and 1 X-linked among the inherited)		12% of diagnosed patients
Johannesen et al. (55)	200 adults	Epilepsy suggestive of a genetic etiology (91% with intellectual disability)	Gene panel: 23%	SNVs in 69% of the diagnosed patients	46% were *de-novo* variants, 9 % inherited from affected parents, rest unknown		17% of diagnosed patients
Zacher et al. (30)	150 adults	Epilepsy and intellectual disability of unknown origin	Fragile X testing: 0.7% Karyotyping: 2% CGH-array: 16% Gene panel: 22.7% WES: an additional 8.7% Trio WES: an additional 2%	SNVs in 69% of the diagnosed patients	36.7% of the SNVs were *de-novo*, 18.4% of SNVs were inherited (4 AR, 2 X-linked, 1 with an SNV inherited via a parental mosaic, and 2 with variants associated with disorders known to be of reduced penetrance, each inherited from healthy parents), rest not defined	Severity of the intellectual disability, febrile seizures and evidence of alleged or unproven exogenic factors	45.1% (11.8% with the highest level of evidence)

*DEEs, developmental and epileptic encephalopathies; WES, whole exome sequencing; SNV, single nucleotide variants; AD, autosomal dominant; AR, autosomal recessive; CGH-array, comparative genomic hybridization-array*.

Minardi et al. (54) presented a series of 71 adult patients with DEEs of unknown etiology, according to the International League Against Epilepsy (ILAE) classification. Of them, 90.1% had already undergone prior genetic testing (karyotype, CGH-array, single gene or gene panel screening), which resulted as negative. WES resulted in a diagnostic yield of 25.3% regarding pathogenic (or likely pathogenic) variants according to the American College of Medical Genetics (ACMG) guidelines. Pathogenic or likely pathogenic variants were significantly more frequent among patients displaying brain MRI malformations, early onset epilepsy or dysmorphisms. In 50% of diagnosed cases, management was directly impacted by the results (mostly by receiving accurate genetic counseling, but also by changes in anti-seizure drugs and monitoring of specific comorbidities).

Benson et al. (37) published a series of 74 adults and 27 pediatric patients with medically refractory epilepsy and comorbid intellectual disability of unknown etiology. Previous testing with gene panels or WES were exclusion criteria, although single gene tests were accepted. Parent-offspring trio WES was performed to the whole sample, and 80/101 patients were also tested with CGH-array. A first selection of qualifying variants was accomplished by a multidisciplinary team, basing on their prevalence, prediction software tools and implications of genes known to cause epilepsy or intellectual disability according to the Online Mendelian Inheritance in Man (OMIM) compendium. Qualifying variants were further classified into (likely) pathogenic according to ACMG guidelines. Pathogenic or likely pathogenic variants were found in 30% of adults. In 12% of all diagnosed patients, the results supposed a clear impact on their epilepsy treatments, by changes in anti-seizure drugs and the opening of further precision therapy settings. Four potential incidental findings not epilepsy-related were also described after WES.

Johannesen et al. (55) reported 200 adult patients with epilepsy referred to a specialized epilepsy center for diagnostic purposes. These patients displayed a medical history particularly suggestive of a genetic etiology, and 91% of them suffered from comorbid intellectual disability. Patients were tested using customized epilepsy gene panels. Most patients were assessed with a panel involving at least 100 genes. Variants were classified according to the ACMG guidelines, and 23% of the cohort was diagnosed with (likely) pathogenic variants. Seventeen percent of diagnosed patients benefited from therapeutic changes directly related with their genetic findings.

Finally, Zacher et al. (30) recently reported a series of 150 adults with epilepsy and intellectual disability (intelligence quotient of 70 or less) of unknown origin. Pathogenicity of variants was assessed according to the ACMG guidelines and ClinGen. Firstly, patients underwent karyotyping, Fragile-X testing, CGH-array and gene panel sequencing, identifying (likely) pathogenic variants in 38% (panel sequencing accounting for 22.7%, CGH-array 16%, karyotyping 2% and Fragile-X-testing 0.7%). Single or parent-offspring trio WES (including coverage-based analysis of CNVs in addition to previous CGH-arrays) was performed in the 93 remaining undiagnosed patients, respectively, diagnosing an additional 8.7 and 2% of (likely) pathogenic variants of the overall cohort. All chromosomal aberrations detected by karyotyping were also detected by CGH-array and NGS techniques, and all CMVs detected by CGH-array were also detected by NGS techniques. Factors correlating with the diagnostic yield were the severity of the intellectual disability, febrile seizures and evidence of alleged or unproven exogenic factors. Almost 12% of the diagnosed patients benefited from precision medicine approaches.

**Figure 1:**
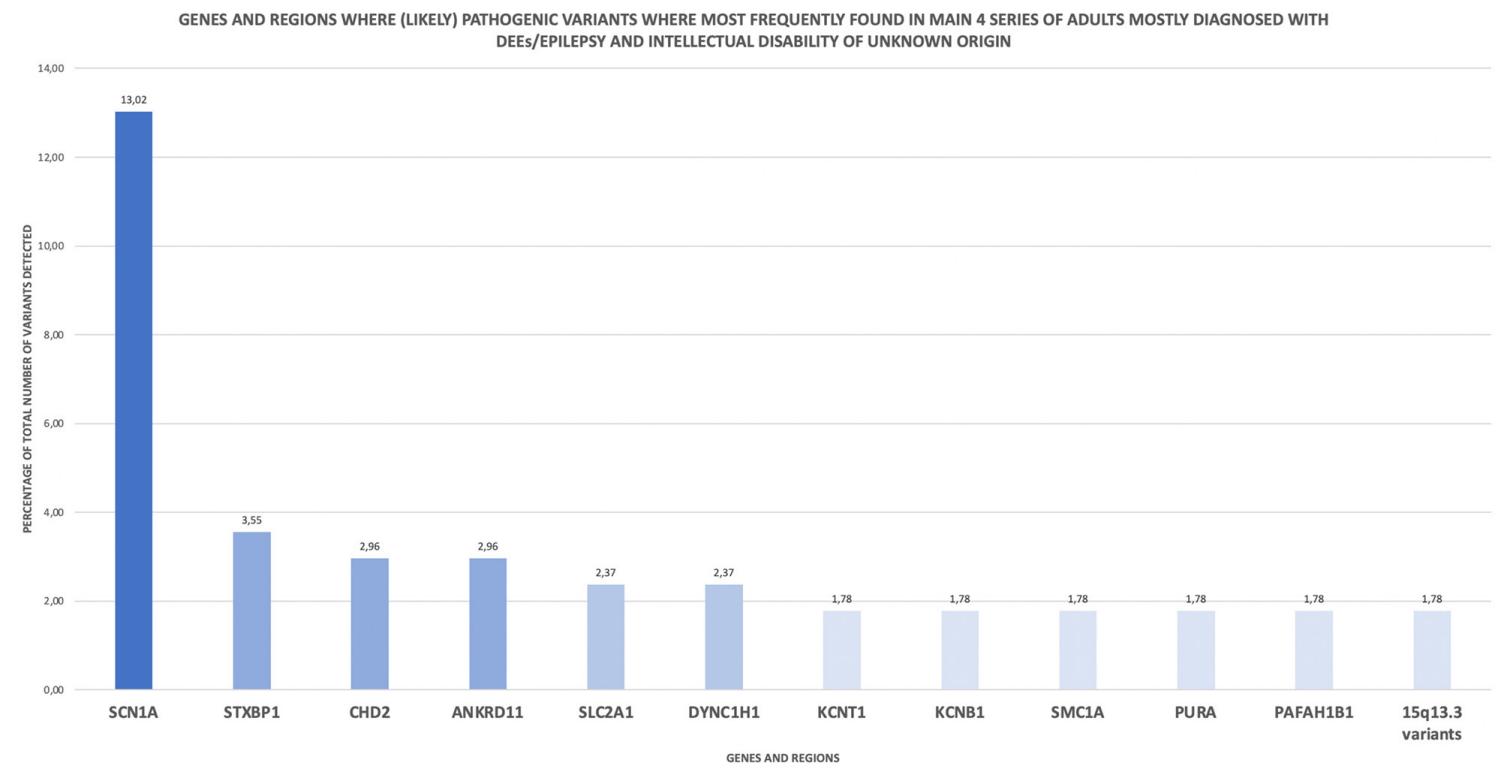
Genes and regions where (likely) pathogenic variants where most frequently found in main 4 series of adults mostly diagnosed with DEEs/epilepsy and intellectual disability of unknown origin. This descriptive analysis is based on the published data of 4 main series of adult patients displaying the mentioned pheynotype (30, 37, 54, 55).

Basing on the data provided by the authors of these four series in their published papers and supporting material, a simple descriptive analysis on the most frequently detected variants can be performed. Of 169 variants detected overall, 37.9% groups in 12 main genes or regions (Figure 1), being SCN1A, STXBP1, CHD2, ANKRD11, SLC2A1, and DYNC1H1 the genes where (likely) pathogenic variants where most frequently found. Among them, SCN1A was the most affected gene, gathering 13% of the reported variants.

Hence, basing on these series and if the initial diagnostic workup is inconclusive, the target group of adult patients meant to be identified for genetic testing, would be those displaying epilepsy (even if focal epilepsy phenotypes) and other neurological or systemic manifestations, especially intellectual disability or other neurodevelopmental disorders (with wide-ranging phenotypes and severity), family history of epilepsy, early onset epilepsy, febrile seizures, large malformations of cortical development or dysmorphisms.

**Figure 2:**
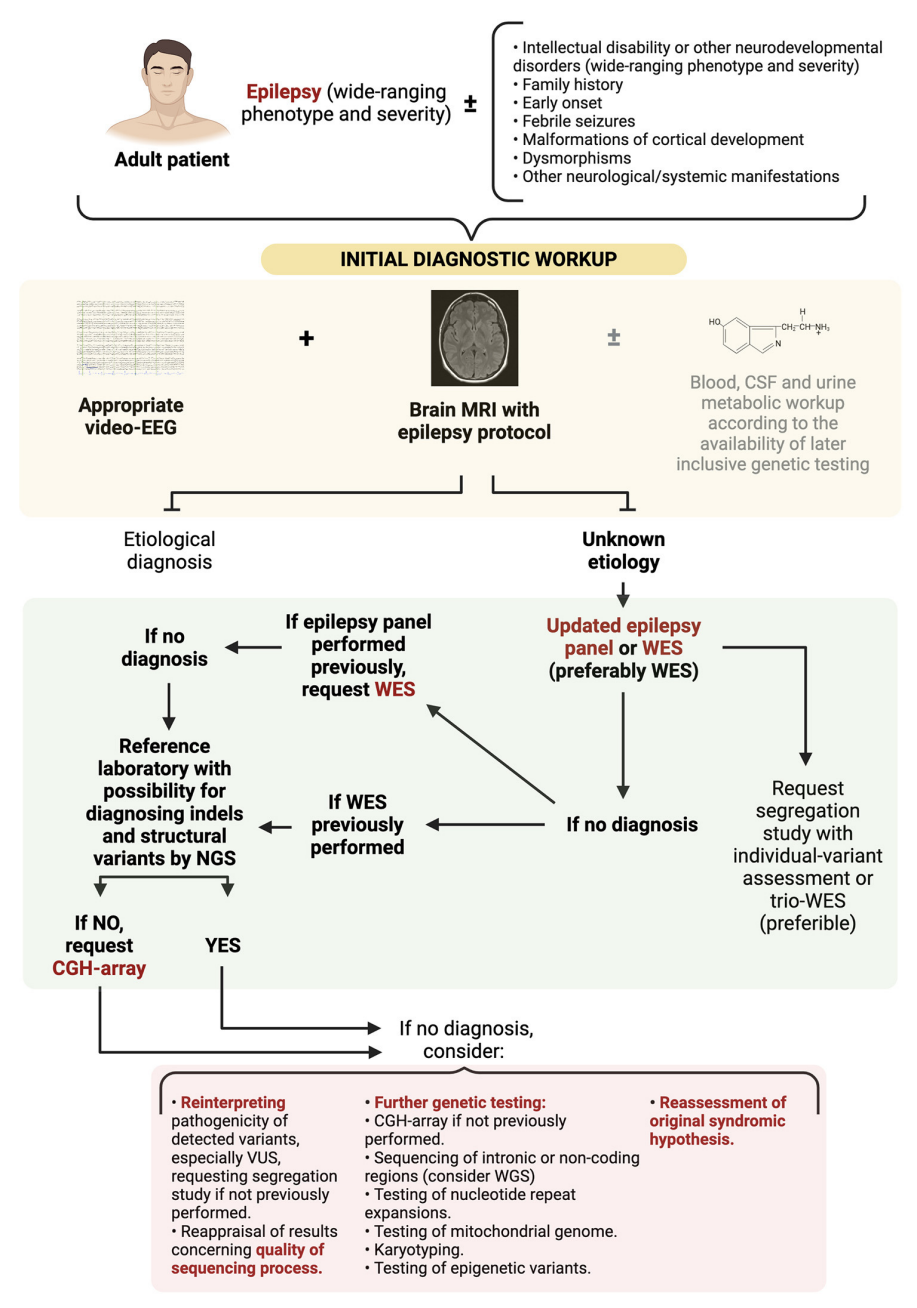
Diagnostic approach to adult patients with suspected DEEs of genetic origin. WES, whole genome sequencing; WGS, whole genome sequencing; CGH-array, comparative genomic hybridization-array; VUS, variants of uncertaing significance.

In any case, it is important to remark that although patients with DEEs represent the vast majority of candidates for genetic testing so far, the concept of genetic epilepsy is wider and extends beyond the DEEs (as illustrated in Figure 2). Thus, the absence of developmental delay or intellectual disability does not exclude the possibility of a genetic origin. For instance, autosomal dominant lateral temporal lobe epilepsy is characterized by focal seizures with onset in adolescence, in patients without intellectual disability or other neurological manifestations, displaying non-lesional brain MRIs. Two or more members of the families show a similar phenotype, and variants in LGI1 gene account for the majority of the familial cases (56).

On the other hand, in adults with epilepsy without any of the above-mentioned complementary features, the probability of obtaining causal variants is lower, and genetic testing would not be indicated from the start within a diagnostic context. Nonetheless, this setting might change in future, if the knowledge of genetic background is able to provide management-changing information regarding treatment or prognosis in epilepsies with mixed etiologies, such as in pre-surgical scenarios (57).

## Which Genetic Test to Perform

Updated recommendations on which genetic test to perform regarding these groups of patients should be based on the still poor genotype-phenotype correlations, the type of variants and the diagnostic yield of the different techniques found in the above-mentioned series. In this sense, it may still not be clinically worthwhile to limit our daily diagnostic efforts to single gene analysis or excessively limited gene panels in the contemporary setting, in order to prevent patients from undergoing an even longer diagnostic odyssey.

The vast majority of variants found in adults with DEEs consisted of *de novo* SNVs and indels affecting exons, followed by CNVs. Modern bioinformatic tools implemented in the context of NGS techniques allow the diagnosis of SNVs, indels, as well as most CNVs and the rest of structural variants, and even mitochondrial genome (the latter two referring particularly to WES and WGS), on the same test and sample, without the need for additional CGH-arrays or karyotyping in most of patients. Thus, first of all, it is useful to be familiar with the concrete covering capacities of the genetic tests performed in our reference laboratory, to assess whether it would be necessary to request the mentioned additional tests to examine CNVs or other structural variants.

Figure 2 illustrates a proposed diagnostic approach algorithm. A first step with an updated epilepsy panel or WES (preferably WES) is warranted. If no significant variants are revealed and a panel had been initially performed, request WES. Instead, if WES had been previously performed and the reference laboratory has no possibility of diagnosing indels and structural variants by NGS, then a CGH-array should be carried out. The cost of WES might represent an obstacle to these initially extensive diagnostic approaches, and lead to a diagnostic gap between settings with different resource availability. Nonetheless, considering its diagnostic yield, there exist initial evidence suggesting that an exome first approach could be more cost-effective and reduce the diagnostic time in individuals with epilepsy of unknown origin, or with rare neurodevelopmental disorders, including those manifesting epilepsy (58, 59). Further investigation is warranted to support this kind of broad initial diagnostic approaches.

Regarding these initial settings, we also suggest considering segregation studies and the implementation of mosaicism-detection techniques. Segregation studies refer to the supplementary genetic testing of the parents (and other relatives, if required) of the proband, in order to facilitate the interpretation of the pathogenicity of the detected variants and the genetic counseling. Parent-offspring trio WES (30) (denoting the performance of WES both to the proband and the parents) may be the best option. If trio WES is not available, at least the suspicious variants observed in the proband should be individually tested in the parents. On the other hand, the depth of the sequencing (>100× in this case) should be checked, so not to mislead a mosaic variant (both in the proband and the parents, if trio WES performed) with absence of anomalies. Mosaic variants represent 5–10% of cases of cases of DEEs of genetic origin wrongly labeled as *de novo* (7), as proved in entities such as *CDKL5* deficiency disorder or *SCN1A*-related Dravet syndrome (38). To this respect, it is necessary to remind that Sanger sequencing, which is still used in many laboratories instead of NGS techniques in segregation studies, does not detect most mosaicisms (60).

If this primary diagnostic strategy is not conclusive (61), a reinterpretation of the pathogenicity of the detected variants (especially those variants of uncertain significance -VUS-) (62) and a reappraisal of the obtained results concerning the quality of the sequencing process (see the next section Considerations on Interpreting the Results of Genetic Testing) is warranted.

Moreover, further genetic tests may be considered, as a way to unveil variants not detectable by comprehensive NGS techniques. Approaches such as karyotyping [diagnosis of ring chromosome 20 or 14 (63), as well as balanced translocations], nucleotide repeat expansions testing (diagnosis of familial cortical myoclonus syndrome, among others), sequencing of mitochondrial genome (if not included previously), sequencing of intronic or non-coding regions *via* WGS, or testing of epigenetic variants, could be considered. Testing of somatic mutations (64), some of them still only used in a research setting, might also be contemplated. Concurrently, a reassessment of the original diagnostic hypothesis should always be exercised in this context.

In addition, based on the literature and on our own clinical experience, the constant 1–2% of unsolicited findings reported in previous series when applying a comprehensive NGS technique such as WES (65), should not discourage its use, since its benefits commonly overcome these circumstances. These include the uncovering of still-undescribed epilepsy-related genes, and variants currently classified as VUS, which might get further pathogenic implications in future, among others.

Nevertheless, there may be exceptions to this prudent and wide diagnostic approach. Clinically well-defined entities such as Dravet, Angelman, tuberous sclerosis complex or Rett syndromes may be more easily recognized and be approached with more directed diagnostic techniques, even initially with single gene sequencing. As previously disclosed, inverse and deep phenotyping frameworks are allowing to break down more and more specific entities, such as PCDH19-related epilepsy, that might also benefit from less-comprehensive genetic testing. In this vein, the appearance of more conditions meeting these criteria is to be expected in the next years.

## Considerations on Interpreting the Results of Genetic Testing

Access to genetic testing is not enough. Not all variants detected will cause disease, even the ones detected within an established DEE gene. Adequate variant interpretation is required. Most clinicians receive the results of genetic testing in the form of a report issued by the genetics laboratory. Within this report, the majority of laboratories include particularizations concerning the quality of the procedures leading to the results, the detected variants, the interpretation of their pathogenicity, and the methodologic strategies accounting for these interpretations, among others.

With regard to technical points, clinicians must carefully check both the type of test performed and, more important, the detailed type of variants not evaluated by the procedure, in order to start figuring out the clinical value of the results. Relevant specifications to be checked are the percentage of bases sequenced > 20x (a reliable number would be more than 99%), and how this coverage concretely distributes along the different regions of the sample, graphically reviewing that the regions of interest have been covered enough. Further technical specifications to be examined are the type of confirmation procedures applied to the detected SNVs (usually Sanger sequencing) and CNVs (usually PCR or MLPA assay), when originally diagnosed by NGS.

In relation to the interpretation of the pathogenicity of variants, most of geneticist assemble their judgement basing on the ACMG guidelines for the interpretation of sequence variants (66). These guidelines delimit 31 criteria (regarding population data, functional outcomes, prediction algorithms, segregation patterns, or allelic information, among others), each with an assigned benignity or pathogenicity and a weight, whose combination results in the classification of the variant into pathogenic, likely pathogenic, VUS, likely benign or benign. Currently, VUS make up the majority of reported variants (67), and are a constant source of misinterpretation and emotional stress for many patients and their families. Overall, guidelines constitute one useful tool upon which a multidisciplinary team is meant to individualize the obtained results taking into account the whole picture.

When results do not show pathogenic or likely pathogenic variants, a specific review of the pathogenicity of VUS is warranted. The same critical thinking could be applied when hastily attributing an etiological condition to a (likely) pathogenic variant (e.g., the relationship of likely pathogenic variants in SCN9A with epilepsy phenotypes is still under discussion) (68).

Along this process, clinicians can contribute the most in deeply assessing the phenotype of the patient and whether or not an association with the target variant is plausible. ACMG criteria are meant to be used as guidelines and are open for interpretation and adaptation. Multiple lines of evidence can be integrated to the criteria to boost interpretation. In this regard, several bioinformatics methods for variant interpretation have been developed (69–71). Other aspects to go over through (the best part included as criteria in the ACMG guidelines) are checking if the target gene has been already related to the phenotype of the patient *via* OMIM or similar databases (72), examining the presence and/or frequency of the target variant in the Genome Aggregation Database (gnomAD) (73) or other general population variant repositories, analyzing if the target variant has been already reported as a disease-related variant in the Human Gene Mutation Database (HGMD) (74), ClinVar (67) or other similar databases, reviewing whether the target variant is located in a highly conserved region of the protein from a phylogenetic point of view, checking if pathogenic variants have previously been described in the same region of the current variant, examining the structural or reading frame effect (truncating or frameshift variants pointing toward pathogenicity), evaluating the results of *in silico* predictors of pathogenicity (PolyPhen, SIFT and MutationTaster, among others), and verifying the consistency with the expected segregation pattern. As will be mentioned later, an assessment of the functional effect (e.g., loss or gain of function) of the identified variant is also warranted in order to guide therapy, especially regarding channelopathies.

Given the multiple methods and scores available for variant interpretation, batch bioinformatic annotation tools have been developed to optimize their integration in a semiautomatic process (75–77).

## Clinical Implications of Diagnosis: Optimization of Therapy and Beyond

Accuracy in diagnosis leads to individualized management approaches, and is in this context where the boundaries of precision medicine have been broadly outlined. Nevertheless, although some major achievements have been reached and future outlooks are promising, clinical implications of genetic diagnosis are in their initial steps, and results for most of patients are still modest. In this context, a review of the pipelines leading to the development of precision therapies and to their effectiveness assessment is warranted in the incoming times (78, 79).

In our opinion, the first and most solid consequences arising from this notion are the ending of a diagnostic odyssey for patients and their families, the information about natural history and prognosis concerning a tangible disease, the accessibility to therapeutic trials in present and future, and advanced genetic counseling when familial segregation has been correctly studied, including complex cases such as parental germinal mosaicism or somatic mutations, and even planning ahead diverse clinical scenarios and comorbidities (80).

In a second step, emerging aimed therapeutic considerations are starting to be delineated for a number of DEE entities, relating to the optimization of indications of known anti-seizure drugs, and repurposing of drugs without epilepsy-related indications. Examples of these strategies are the possible favorable response to specific antiseizure medications (*KCNQ2* DEE and sodium channel blockers), other families of drugs (*KCNA2* DEE with gain of function and aminopyridine) or dietary treatments (*SLC2A1* and ketogenic diet), the avoidance of specifically harmful drugs in some entities (*POLG* encephalopathies and valproic acid) (6, 81–83), or the possible tendency to relapse manifested by patients with other DEEs when discontinuing anti-seizure medication after a long seizure-free period (*PCDH19* DEE), among many other examples (84, 85).

Finally, throughout the more than 1,200 genes that may be linked to epilepsy-related phenotypes according to the OMIM compendium, just a few tens of them can benefit from further etiopathology-guided therapeutic approaches, being their ultimate representation the ongoing antisense oligonucleotide trials for patients with Dravet syndrome related to specific SCN1A variants, and other new advanced small molecules and gene therapies in the horizon for this and other genetic etiologies in the near future (86).

**Figure 3:**
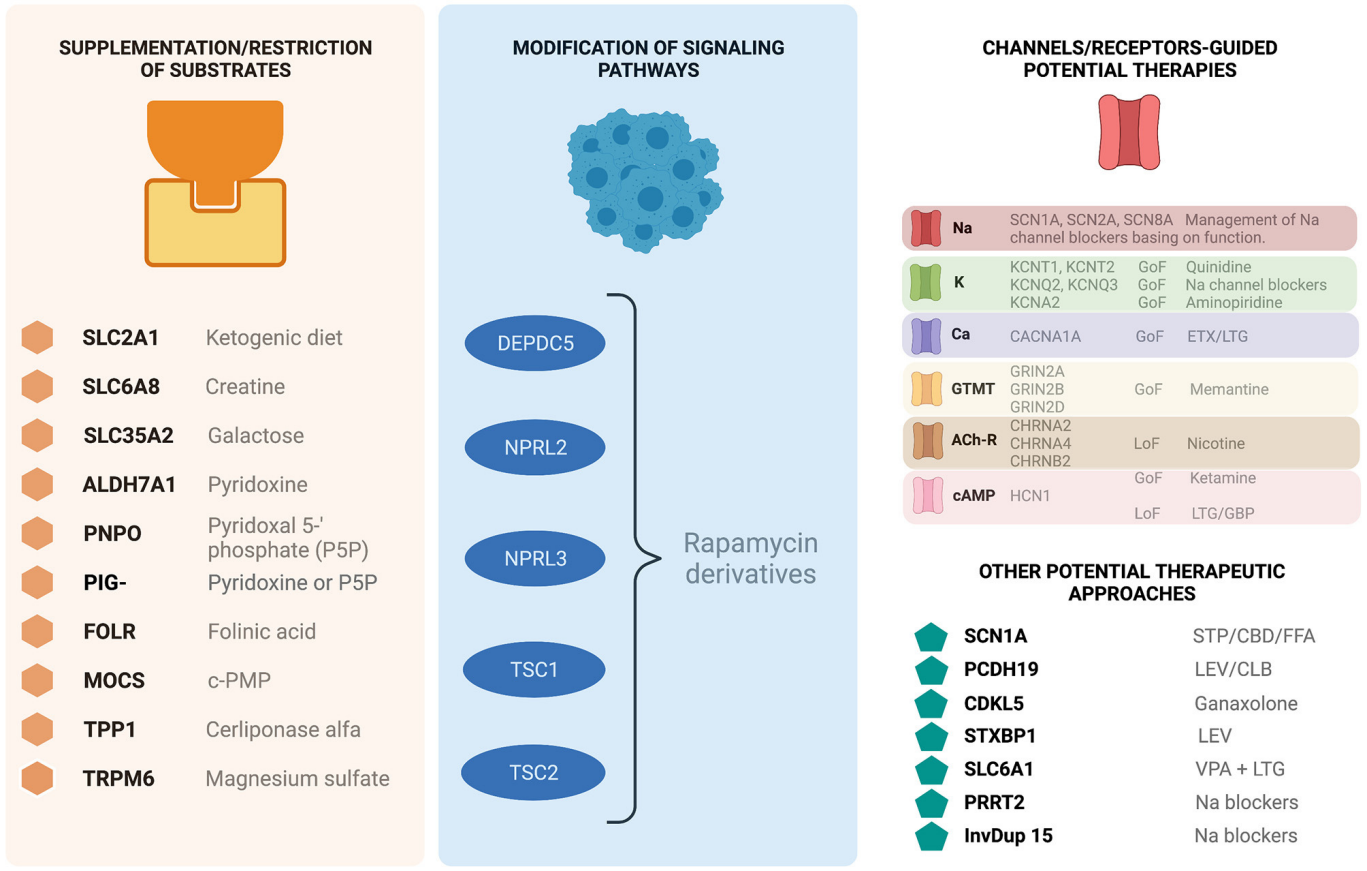
Epilepsy-related genetic conditions displaying potential specific therapeutic approaches in a broadly-defined precision medicine context. GoF, gain of function; LoF, Loss of Function; ETX, ethosuximide; LTG, lamotrigine; GBP, gabapentine; STP, stiripentol; CBD, cannabidiol; FFA, fenfluramine; LEV, levetiracetam; CLB, clobazam; VPA, valproic acid; CBZ, carbamazepine. Modified from Nabbout and Kuchenbuch (6) and Bayat et al. (81).

Most of these monogenic epilepsies have a pediatric presentation, but as introduced earlier, many patients reach adulthood still suffering a diagnostic gap and there are even adult-onset cases reported in literature (87). This sort of specific therapeutic approaches may lay upon more or less solid evidence foundation and may have been reported as more or less clinically effective. It is beyond the scope of this review to describe in detail each one of the reported variants and their corresponding management strategies in this setting. Literature is being constantly updated and its regular re-examination will be necessary. Nabbout and Kuchenbuch (6) conceptualize these therapies in three groups: (1) those concerning the supplementation or restriction of substrates, (2) those concerning therapies modifying signaling pathways, and (3) those concerning therapies modifying channel function in channelopathies (Figure 3 depicts a summarizing diagram of the three groups).

To conclude, a proper diagnostic approach, the extraction of reliable results and a right interpretation of them are worthy for the purpose of reaching the above-mentioned management implications, (concluding the diagnostic odyssey, giving genetic counseling, improving therapy, joining support specific support groups) eventually leading to an improvement in the quality of life of our patients.

## Author Contributions

ÁB-C, ÁA-S, EP-P, and AG-N: ideation, structure, and development of contents, and literature review. ÁB-C, ÁA-S, and AG-N: writing of the draft. ÁB-C, ÁA-S, RM, EP-P, AG-N, IG-M, and RT: review of the draft. All authors contributed to the article and approved the submitted version.

## Conflict of Interest

The authors declare that the research was conducted in the absence of any commercial or financial relationships that could be construed as a potential conflict of interest.

## Publisher's Note

All claims expressed in this article are solely those of the authors and do not necessarily represent those of their affiliated organizations, or those of the publisher, the editors and the reviewers. Any product that may be evaluated in this article, or claim that may be made by its manufacturer, is not guaranteed or endorsed by the publisher.
